# T-bet^+^CD11c^+^ B cells are critical for antichromatin immunoglobulin G production in the development of lupus

**DOI:** 10.1186/s13075-017-1438-2

**Published:** 2017-10-05

**Authors:** Ya Liu, Shiyu Zhou, Jie Qian, Yan Wang, Xiang Yu, Dai Dai, Min Dai, Lingling Wu, Zhuojun Liao, Zhixin Xue, Jiehua Wang, Goujun Hou, Jianyang Ma, John B. Harley, Yuanjia Tang, Nan Shen

**Affiliations:** 1 Shanghai Institute of Rheumatology, Renji Hospital, School of Medicine, Shanghai Jiao Tong University, 145 Shan Dong Middle Road, Shanghai, 200001 China; 2 Institute of Health Sciences, Shanghai Institutes for Biological Sciences (SIBS) & Shanghai Jiao Tong University School of Medicine (SJTUSM), Chinese Academy of Sciences (CAS), Shanghai, China; 3 Cincinnati Children’s Hospital Medical Center, University of Cincinnati College of Medicine, and Cincinnati VA Medical Center, Cincinnati, Ohio USA; 4 Center for Autoimmune Genomics and Etiology (CAGE), Cincinnati Children’s Hospital Medical Center, Cincinnati, Ohio USA; 5 State Key Laboratory of Oncogenes and Related Genes, Shanghai Cancer Institute, Renji Hospital, Shanghai Jiao tong University School of Medicine, Shanghai, China

**Keywords:** Systemic lupus erythematosus, Chronic graft-versus-host disease, B cell, Antichromatin antibody, T-bet

## Abstract

**Background:**

A hallmark of systemic lupus erythematosus is high titers of circulating autoantibodies. Recently, a novel CD11c^+^ B-cell subset has been identified that is critical for the development of autoimmunity. However, the role of CD11c^+^ B cells in the development of lupus is unclear. Chronic graft-versus-host disease (cGVHD) is a lupus-like syndrome with high autoantibody production. The purpose of this study was to explore the role of CD11c^+^ B cells in the pathogenesis of lupus in cGVHD mice.

**Methods:**

cGVHD was induced by an intraperitoneal injection of 5 × 10^7^ Bm12 splenocytes into B6 mice. Flow cytometry was used to analyze mice splenocytes and human samples. Magnetic beads were used to isolate mice B cells. Gene expression was determined by real-time quantitative polymerase chain reaction (RT-qPCR). Enzyme-linked immunosorbent assay (ELISA) was used to detect antibodies in serum and supernatants.

**Results:**

The percentage and absolute number of CD11c^+^ B cells was increased in cGVHD-induced lupus, with elevated levels of antichromatin immunoglobulin (Ig)G and IgG2a in sera. CD11c^+^ plasma cells from cGVHD mice produced large amounts of antichromatin IgG2a upon stimulation. Depletion of CD11c^+^ B cells reduced antichromatin IgG and IgG2a production. T-bet was upregulated in CD11c^+^ B cells. Knockout of T-bet in B cells alleviated cGVHD-induced lupus. Importantly, the percentage of T-bet^+^CD11c^+^ B cells increased in lupus patients and positively correlated with serum antichromatin levels.

**Conclusion:**

T-bet^+^CD11c^+^ B cells promoted high antichromatin IgG production in the lupus-like disease model cGVHD. In lupus patients, the percentage of T-bet^+^CD11c^+^ B cells was elevated and positively correlated with antichromatin antibodies. The findings provide potential therapeutic insight into lupus disease treatment.

**Electronic supplementary material:**

The online version of this article (doi:10.1186/s13075-017-1438-2) contains supplementary material, which is available to authorized users.

## Background

Systemic lupus erythematosus (SLE) is a prototypic autoimmune disease characterized by an array of autoantibodies that target multiple normal cellular components [1–3]. When encountered by self-antigens, autoantibodies can bind with them to form immunoglobulin complexes (ICs) which deposit in the kidney, activate the complement system, and trigger a series of inflammatory responses. Accumulating evidence has indicated that the presence of certain autoantibodies are highly associated with some symptoms of the disease [4, 5]. For example, antiphospholipid antibodies are critically linked to the development of thrombotic events and obstetric morbidity [6], antiribonucleoprotein (RNP) antibodies are associated with myositis and Raynaud’s phenomenon [7], and antidouble-stranded DNA (dsDNA) antibodies are associated with lupus nephritis [8]. However, the characterization of B cells that produce these distinct kinds of pathogenic antibodies is still unclear.

Chromatin, the native complex of histones and DNA found in the cell nucleus of eukaryotes, consists of approximately 40% DNA, 40% histones, 20% nonhistone proteins, RNA, and other macromolecules [9]. Data accumulated over recent years have indicated that antichromatin autoantibody is involved in the pathogenesis of SLE and is associated with disease activity and lupus nephritis [10–12]. Several studies of lupus-like murine models have found that genetic loci, such as sle1, are related to antichromatin antibody production [13]. Immunization with active chromatin induces lupus-like syndrome in BALB/c mice [14]. Certain knockout mice, such as C1q, serum amyloid protein (SAP) and Dnase I-deficient mice, have high titers of antichromatin antibodies [15, 16]. However, it is not quite clear how B cells lose tolerance to chromatin in the development of lupus.

Transfer of major histocompatibility complex (MHC) II-mismatched splenocytes from Bm12 mice into B6 mice causes a chronic graft-versus-host disease (cGVHD) [17–19]. The majority of autoantibodies in cGVHD are antichromatin and anti-erythrocytes [20]. Recently, a novel B-cell subset, CD19^+^CD11c^+^, has been identified in aged female B6 mice that is able to produce large amounts of antichromatin autoantibody in response to the Toll-like receptor (TLR)7 ligand in vitro [21]. However, little is known about the differentiation and function of CD19^+^CD11c^+^ B cells in the development of lupus.

In this manuscript, we examined the role of CD19^+^CD11c^+^ B cells in the development of lupus induced by cGVHD. Our data showed that CD11c^+^ B cells were able to produce large amounts of antichromatin autoantibody, in particular the immunoglobulin (Ig)G2a isotype, in cGVHD mice. T-bet was critical for CD11c^+^ B-cell development and antichromatin autoantibody production. Finally, we analyzed the phenotype of T-bet^+^CD11c^+^ B cells in lupus patients, as well as its correlation with circulating levels of autoantibody.

## Methods

### Human study subject samples

Twenty-two patients with SLE and 10 age-matched and sex-matched normal controls were recruited to analyze the percentage of T-bet^+^CD11c^+^CD19^+^ B cells in peripheral blood mononuclear cells (PBMCs). Healthy donors had no history of autoimmune disease or any treatment with immunosuppressive agents. Patients with concurrent infection were excluded from the study. All SLE patients were recruited from the Renji Hospital and fulfilled the American College of Rheumatology (ACR) 1982 revised criteria for SLE [22], and 14 of these patients met the ACR criteria for lupus nephritis [23]. The Systemic Lupus Erythematosus Disease Activity Index (SLEDAI) score was determined for each patient at the time of blood draw [24]. Additional clinical information about the subjects is listed in Table 1. Informed consent was obtained from all the subjects. The study was approved by the Research Ethics Board of Shanghai Renji Hospital.

**Table 1:** Demographic data

Characteristic	SLE patients (*n* = 22)	Healthy donors (*n* = 10)
Sex (male/female)	0/22	0/10
Age, years (mean ± SD)	36.68 ± 11.03	39.00 ± 12.42
Disease duration, months (mean ± SD)	74.59 ± 26.46	–
SLEDAI score (mean ± SD)	9.00 ± 4.163	–
Anti-ANA (positive/negative)^a^	20/0	–
Lupus nephritis (positive/negative)	14/8	–
Proteinuria (positive/negative)	10/12	–

^a^Not all patients were evaluated

*ANA*, antinuclear antibody, *SLE* systemic lupus erythematosus, *SLEDAI* Systemic Lupus Erythematosus Disease Activity Index

### Mice

B6(C)-H2-Ab1bm12/KhEgJ(Bm12), C57BL/6 J(B6), B6.129P2-Igh-Jtm1Cgn/J (μMT), B6.FVB-Tg(Itgax-DTR/EGFP)57Lan/J(B6.CD11c-DTR), and B6.129S7-Ifngr1tm1Agt/J (B6.IFNGR1^–/–^) were purchased from The Jackson Laboratory (Bar Harbor, ME). The bm12 strain differs from the B6 by three amino acids in the beta chain of the I-A molecule [25]. Bm12 and B6 were propagated in the animal facility at Cincinnati Children’s Hospital Medical Center (CCHMC; Cincinnati, USA). μMT and B6.CD11c-DTR mice were maintained in the animal facility at the Institute of Health Science (IHS; Shanghai, China). All animals were 10–12 weeks old at the time of experimentation. All animal protocols were approved by the Animal Care and Use Committee of CCHMC and IHS.

### cGVHD induction

A single-cell suspension of Bm12 splenocytes was prepared in 1× phosphate-buffered saline (PBS) and filtered through 0.2-μm sterile nylon mesh; 5 × 10^7^ splenocytes were then intraperitoneally injected into B6 mice. After 2 weeks, the recipient mice were sacrificed for analysis.

### Antibodies and flow cytometry

The following monoclonal antibodies (mAbs) used for staining were purchased from BioLegend/BD bioscience: APC-Cy7 anti-CD19, BV421 anti-CD138, APC-eF780 anti-CD11c, Bv605 anti-CD19, FITC anti-CD11c, PE anti-IgG2a, Bv421 anti-CD4, APC anti-IFNγ, and APC anti-T-bet. eF506 Live/Dead dye was obtained from eBioscience. Cells were fixed in BD Cytofix™ buffer (BD bioscience) before FACS analysis. Intracellular staining for T-bet was performed using the BD Cytofix/Cytoperm™ Kit (BD bioscience). Data were collected on Fortessa2 and an LSR-II flow cytometer and analyzed by FlowJo software.

### Cell isolation and in vitro culture

Spleen cells from cGVHD mice were pooled together (*n* = 5). CD19^+^ B cells were first positively selected using CD19-positive MACS beads. CD11c^+^CD138^+^ and CD11c^–^CD138^+^ cells were then sorted by FACSDiva. Equal cell numbers were then cultured in RPMI-1640 supplemented with 10% fetal bovine serum (FBS), 2 mM l-glutamine, 100 U/ml penicillin, 100 U/ml streptomycin, and 50 μM 2-mercaptoethanol for 72 h. Lipopolysaccharide (LPS; 1 μg/ml) or R848 (1 μg/ml) were used for cell stimulation. The supernatant was assayed by antichromatin antibody enzyme-linked immunosorbent assay (ELISA). To test for interferon (IFN)γ^+^ T cells, 2 × 10^7^ spleen cells from cGVHD mice were cultured in the above cell culture medium with 1× cell stimulation cocktail (plus protein transport inhibitors) (eBioscience) for 6 h and then stained with Live/Dead dye, anti-CD4, and anti-IFN-γ mAbs.

### ELISA for antichromatin antibodies

Chromatin was prepared as previously described [26, 27]. Plates were coated with chromatin at 3 μg/ml overnight at 4 °C. The plates were then washed and incubated with blocking buffer for 2 h at room temperature (RT). The plates were washed again and then 1/500 diluted serum samples were added in duplicate and incubated for 2 h at RT. Biotin-labeled anti-IgG, anti-IgG1, anti-IgG2a, anti-IgG2b, and IgG3 (BioLegend) were used as capture antibodies, and streptavidin-conjugated horseradish peroxidase (HRP) (Thermo Scientific) was used as the detective antibody. Tretramethylbenzidine (TMB, 1×; eBioscience) was then added and incubated for 30 min at RT. Sulfuric acid (2 N) was used as a stop solution and optical density (OD) values were then measured at 450 nm and 570 nm.

Sera antichromatin and anti-dsDNA antibodies of SLE patients were measured with ELISA kits from Inova Diagnostics Company. The experiments were performed according to the manufacturer’s instructions.

### Depletion of CD11c^+^ B cell in cGVHD mice

CD11c-DTR mice with cGVHD were induced by transferring 5 × 10^7^ splenocytes from Bm12. For depletion of CD11c^+^ cells, mice were then intraperitoneally injected with 100 ng diphtheria toxin (in PBS; Sigma) at day 7, day 9, and day 11 post-cGVHD induction. The efficiency of depletion was then examined by flow cytometry 14 day post-cGVHD induction.

### Statistical analysis

Data were analyzed using GraphPad Prism (version 5.01). Statistical differences were calculated using one-way analysis of variance (ANOVA) and the Student’s *t* test. Nonparametric correlation (Spearman) was used for correlation studies. Values are presented as the mean ± standard deviation (SD). A value of *p* < 0.05 was considered to be statistically significant.

## Results

### CD11c^+^ B cells were increased in cGVHD autoimmune mice

**Fig. 1:**
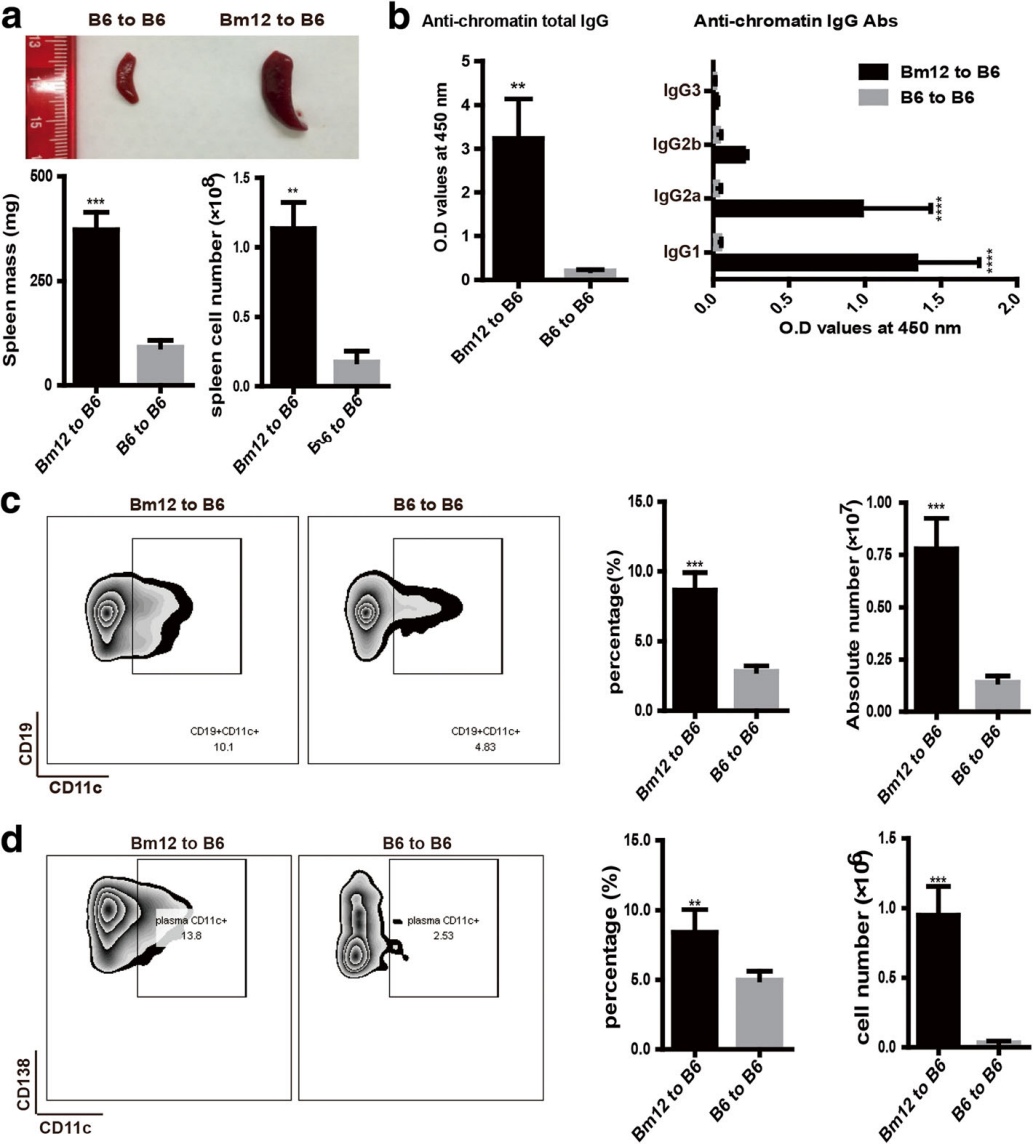
CD11c^+^ B cells were increased in cGVHD mice. B6 mice (*n* = 5) received an intraperitoneal injection of 5 × 10^7^ splenocytes from Bm12 or B6 mice; spleens and serum were collected at day 14 for flow analysis and the antibodies examination. **a** Weight and cell counts of the spleen were taken 14 days later. The figure shows spleen photographs, spleen weight, and absolute number of splenocytes. **b** Serum antichromatin total IgG and IgG subtype titers were examined by ELISA. **c**, **d** Flow cytometric analysis of CD11c^+^CD19^+^ cells (**c**) and CD11c^+^CD138^+^ cells (**d**) in total spleen. Values are shown as the mean ± SD. **P < 0.01, ***P < 0.001, ****P < 0.0001. *Abs* antibodies, *IgG* immunoglobulin G, *OD* optical density

Consistent with the results of the earlier study [20], we found that B6 mice that received splenocytes from Bm12 mice developed a lupus-like syndrome after 14 days of injection. As illustrated in Fig. 1a and b, both the mass and cell number of the spleen in the Bm12 to B6 group were elevated along with higher levels of antichromatin IgG and IgG2a antibodies in the sera compared with the B6 to B6 group. It has been reported that the CD11c^+^ B-cell population accumulated in both old female mice and humans with autoimmune disease, and might play a direct role in the development of autoimmunity [21]. Thus, we next analyzed the phenotype of CD11c^+^ B cells by flow cytometry in cGVHD mice. Our data showed that CD19^+^CD11c^+^ B cells were dramatically increased during the development of cGVHD (Fig. 1c). Furthermore, the fraction of the CD11c^+^ population in CD138^+^ plasma cells was also markedly elevated in cGVHD mice (Fig. 1d). Based on the above evidence, we speculated that CD11c^+^ B cells were probably involved in the development of lupus by autoantibody production.

### CD11c^+^ plasma cells produced large amounts of antichromatin IgG in vitro

**Fig. 2:**
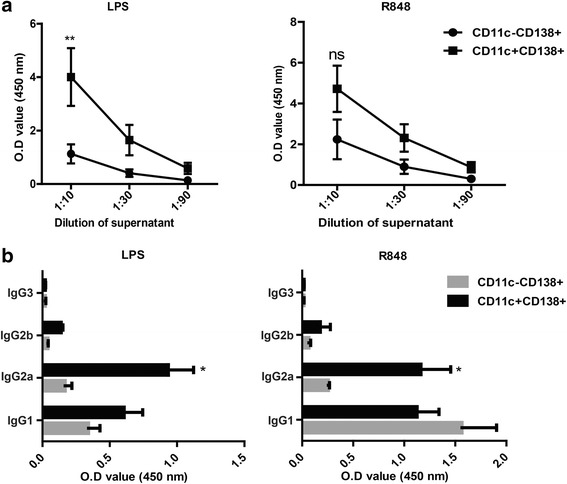
CD11c^+^ plasma cells in cGVHD mice produced antichromatin antibodies after stimulation in vitro. CD11c^+^CD138^+^ and CD11c^–^CD138^+^ cells were sorted from cGVHD mice and cultured for 7 days in the presence of the TLR4 (lipopolysaccharide; *LPS*) or TLR7 agonist (R848). Antichromatin total IgG (**a**) and antichromatin IgG subclass (**b**) in the supernatant were subsequently measured by ELISA. In (**a**), the *x*-axis shows the dilution factors. Bars represent mean (± SD) of three independent experiments. *P < 0.05, **P < 0.01. *IgG* immunoglobulin G, *ns* not significant, *OD* optical density

To further investigate the role of CD11c^+^ cells in cGVHD-induced lupus, we sorted CD11c^+^CD138^+^ and CD11c^–^CD138^+^ cells from mice that received Bm12 splenocytes and performed an in vitro functional assay. As indicated in Fig. 2a, CD11c^+^CD138^+^ cells produced more antichromatin IgG antibodies than did CD11c^–^CD138^+^ cells in response to LPS or R848 stimulation, although no statistical significance was observed in the R848 group.

The effector mechanisms of the subclasses of antibodies were distinct due to different constant regions. IgG2a is reported to have the most protective and pathogenic properties among mouse IgG subclasses [28, 29]. Notably, we found that antichromatin IgG2a was exclusively produced by CD11c^+^CD138^+^ cells (Fig. 2b).

### Depletion of CD11c^+^ B cells ameliorated antichromatin IgG production in vivo

**Fig. 3:**
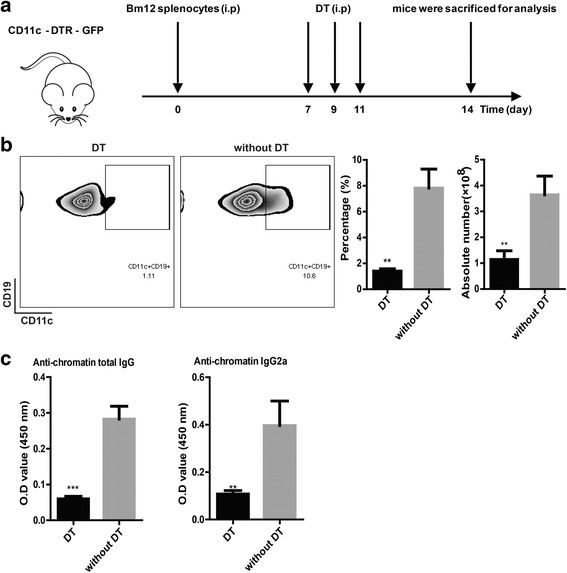
In vivo depletion of CD11c^+^ B cells attenuated the production of antichromatin IgG after cGVHD induction. **a** Design of transient depletion of CD11c^+^ B cells in the cGVHD study. Mice received three intraperitoneal (*i.p.*) injections of 100 ng diphtheria toxin (*DT*) (*n* = 5) or negative control (PBS; *n* = 5) every other day after 7 days from an intraperitoneal injection of splenocytes from Bm12; after 14 days, the recipient mice were sacrificed for analysis. **b** Flow cytometric analysis of CD19^+^CD11c^+^ cells in total spleen. **c** Antichromatin IgG and IgG2a autoantibodies were measured by ELISA in the serum of CD11c-DTR mice with cGVHD induction. Results are representative of three independent experiments. Values are shown as the mean ± SD. **P < 0.01, ***P < 0.001. *IgG* immunoglobulin G, *OD* optical density

Next, we wanted to know whether depletion of CD11c^+^ B cells in cGVHD mice could reduce the level of antichromatin IgG in vivo. To this end, CD11c-DTR mice were transferred with 5 × 10^7^ splenocytes of Bm12. The mice then received an intraperitoneal injection of 100 ng diphtheria toxin at day 7, day 9, and day 11 (Fig. 3a). As expected, the percentage and absolute number of CD11c^+^ B cells was dramatically reduced by diphtheria treatment in CD11c-DTR mice that received Bm12 splenocytes (Fig. 3b). Moreover, transient depletion of CD11c^+^ B cells significantly decreased the circulating levels of antichromatin IgG and IgG2a antibodies (Fig. 3c). In general, these results demonstrated that CD11c^+^ B cells might be critical for antichromatin IgG production, both in vitro and in vivo.

### T-bet^+^CD11c^+^ B cells were significantly increased after cGVHD induction

**Fig. 4:**
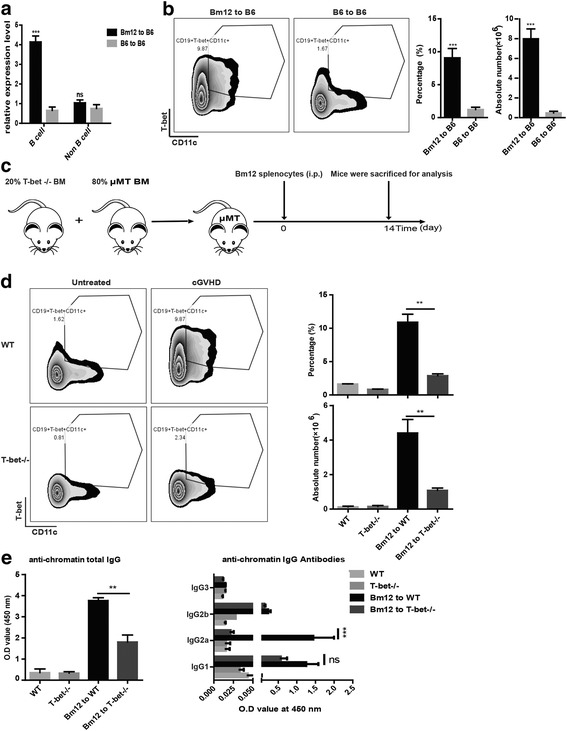
Antichromatin IgG production during cGVHD required T-bet^+^CD11c^+^CD19^+^ B cells. **a** CD19^+^ cells and CD19^–^ cells of the spleen were isolated from B6 mice transferred with splenocytes from Bm12 or B6 mice at 14 days to examine T-bet expression. **b** Flow cytometric analysis of T-bet^+^CD11c^+^CD19^+^ B cells in the total spleen at day 14 with chronic graft-versus-host disease (*cGVHD*) induction. **c** Design of the T-bet-deficient B-cell study. B cells of knockout T-bet were transferred into μMT mice (*n* = 10). The mice were then intraperitoneally (*i.p.*) injected with 5 × 10^7^ Bm12 splenocytes. Spleen and serum were collected at 14 days after cGVHD induction. **d** Flow cytometric analysis of T-bet^+^CD11c^+^CD19^+^ B cells in the total spleen. **e** ELISA was used to measure the production of sera antichromatin IgG and IgG subclass. Results in (**a**) are representative of three independent experiments. Values are shown as the mean ± SD. **P < 0.01, ***P < 0.001. *IgG* immunoglobulin G, *ns* not significant, *BM* bone marrow, *OD* optical density, *WT* wild-type

IgG2a is known as the most pathogenic antibody in autoimmune disease [30, 31]. Many studies have demonstrated that T-bet is critical for IgG2a class switching [32, 33] and IgG2a memory response [34]. Recently, it has been reported that T-bet drives CD11c^+^ B-cell activation to secrete a virus-specific IgG2a antibody upon virus infection [35]. Thus, we next investigated the role of T-bet in CD11c^+^ B-cell differentiation and antichromatin IgG2a production in cGVHD-induced lupus. As shown by quantitative polymerase chain reaction (qPCR), T-bet expression was remarkably upregulated in spleen B cells from B6 mice that received Bm12 splenocytes at 14 days, relative to B6 to B6 mice. However, no significant change was observed in non-B cells (Fig. 4a). Furthermore, the mice that received Bm12 splenocytes showed more T-bet^+^CD11c^+^CD19^+^ B cells than those that received B6 splenocytes (Fig. 4b). As mentioned earlier, CD138^+^ plasma cells were dramatically expanded in cGVHD mice. Notably, we found that more than 70% of CD138^+^ cells were IgG2a-positive (Additional file 1: Figure S1A). The fraction of T-bet^+^ and CD11c^+^ cells was significantly increased in CD138^+^ plasma cells (Additional file 1: Figure S1B). Therefore, these results suggested that T-bet expression might contribute to the production of antichromatin IgG2a in the development of cGVHD.

### Depletion of T-bet^+^ B cells ameliorated antichromatin IgG production in vivo

Next, we wondered whether depletion of T-bet^+^ B cells in cGVHD mice could reduce the level of antichromatin IgG in vivo. We generated B-cell specific T-bet^–/–^ mice by transplanting 20% T-bet-deficient B-cell bone marrow with 80% μMT bone marrow into irradiated μMT mice and then induced cGVHD (Fig. 4c). As expected, depletion of T-bet^+^ B cells inhibited the expression of CD11c in B cells after cGVHD induction (Fig. 4d). Moreover, the levels of antichromatin IgG and IgG2a were significantly decreased in the absence of T-bet^+^ B cells (Fig. 4e). Taken together, our data demonstrated that T-bet is critical for CD11c^+^ B-cell differentiation and antichromatin IgG2a production in cGVHD-induced lupus.

### T-bet^+^CD11c^+^CD19^+^ B cells were significantly increased in lupus patients

**Fig. 5:**
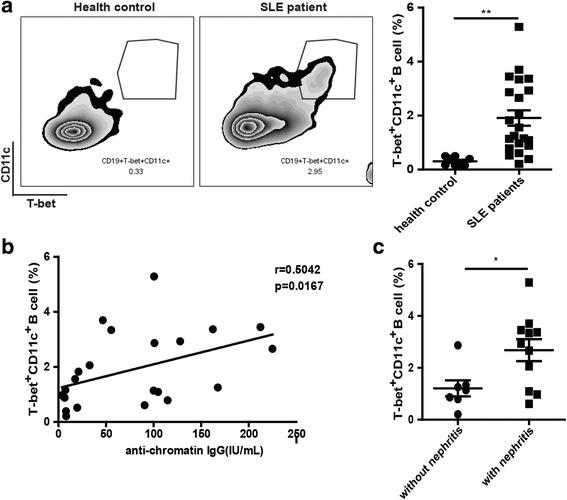
The percentage of T-bet^+^CD11c^+^CD19^+^ B cells was elevated and associated with antichromatin autoantibody in SLE patients. **a** Flow cytometric analysis of T-bet^+^CD11c^+^CD19^+^ B cells in peripheral blood from systemic lupus erythematous (*SLE*) patients (*n* = 22) and healthy donors (*n* = 10). **b** Correlation between the percentage of T-bet^+^CD11c^+^CD19^+^ B cells and the level of antichromatin autoantibody. **c** The percentage of T-bet^+^CD11c^+^CD19^+^ B cells exhibited an increasing trend in patients with lupus nephritis (*n* = 14) relative to patients with no history of lupus nephritis (*n* = 8). In (**a**) and (**c**) values are shown as the mean ± SD. *P < 0.05; **P < 0.01. Symbols (filled circles and squares) represent individual subjects. *IgG* immunoglobulin G

Next, we investigated the abnormality of T-bet^+^CD11c^+^CD19^+^ B cells in lupus patients. As shown in Fig. 5a, the frequency of T-bet^+^CD11c^+^CD19^+^ B cells was significantly increased in the PBMCs of lupus patients. We then analyzed the relationship between the frequency of T-bet^+^CD11c^+^CD19^+^ B cells and autoantibodies in the same group of SLE patients. No significant correlation was observed between the percentage of T-bet^+^CD11c^+^CD19^+^ B cells and the presence of antinuclear antibodies (ANA) and anti-dsDNA antibodies (Additional file 1: Figure S2). However, the proportion of T-bet^+^CD11c^+^CD19^+^ B cells positively correlated with the serum titer of antichromatin IgG in SLE patients (Fig. 5b). Moreover, the percentage of T-bet^+^CD11c^+^CD19^+^ B cells was relatively higher in patients with nephritis than in those without nephritis (Fig. 5c). Our data indicated that T-bet^+^CD11c^+^CD19^+^ B cells might be responsible for the abnormal levels of antichromatin in SLE patients which might prove helpful in the diagnosis and treatment of SLE.

## Discussion

Bm12-induced cGVHD is initiated with injection of MHC II-incompatible spleen cells and leads to a chronic syndrome which closely resembles SLE [17]. This syndrome is characterized by the production of a spectrum of autoantibodies similar to those seen in human SLE, including autoantibodies to erythrocytes, chromatin, nuclear antigens, and, to a more limited degree, dsDNA [36]. It is also accompanied by some of the pathological manifestations of SLE, such as lymphoid hyperplasia and immune complex glomerulonephritis [18, 20]. This model has been demonstrated to be valuable for elucidating the immunopathophysiology of SLE [17, 37, 38].

The typical feature of immunological defects in SLE is the production of autoantibodies [3]. Although autoantibodies were found in SLE more than 60 years ago, more studies need to be performed to illuminate the specific function of an individual autoantibody in the development of lupus [39]. Accumulating evidence has indicated that the detection of certain autoantibodies is essential for the diagnosis and treatment of SLE [40, 41]. Since an antibody with a unique specificity is produced by the corresponding B-cell clone, it is crucial to identify the phenotype of the B cell that produces the pathogenic autoantibody.

Chromatin, the native histone-DNA complex, is found in the nucleus of eukaryotic cells. In recent years, numerous studies have found that antichromatin antibodies are quite specific for SLE [10, 42]. Studies from mice experiments including (NZB × NZW) F1, MRL/lpr mice, and cGVHD mice revealed that antichromatin antibodies play a key role in the pathogenesis of lupus [16, 43, 44]. Recently, it is reported that CD11c^+^ B cells could produce antichromatin autoantibody upon TLR7 stimulation [21]. In this study, we found that CD11c^+^ B cells with a high frequency in cGVHD mice produced huge amounts of antichromatin IgG and IgG2a, implying that these B cells might be involved in the development of lupus. In addition, the percentage and absolute number of CD11c^+^ B cells and the levels of antichromatin IgG and IgG2a antibodies were dramatically reduced by diphtheria treatment in CD11c-DTR mice induced by cGVHD. Thus, we infer that CD11c^+^ B cells play an essential role in antichromatin antibody production. Further studies are needed to exclude the contribution of CD11c^+^ DC to the disease in the DTR experiment.

Early studies have shown that T-bet expression in the B lineage promotes class switching to IgG2a [33] and is indispensable for IgG2a memory formation [45]. Recently, Rubtsova et al. revealed that T-bet^+^CD11c^+^ B cells appear at the peak of the antiviral response, secrete antiviral IgG2a, and are essential for effective viral clearance [35]. IgG2a is the most potent isotype in mediating the development of lupus [46–48]. In our study, we demonstrated that T-bet expression was upregulated in splenic B cells and CD11c^+^ B cells of lupus-like cGVHD mice, with elevated levels of antichromatin IgG and IgG2a in the serum. Interestingly, over 70% of the increased CD138^+^ cells in cGVHD mice were IgG2a-positive. Moreover, T-bet^+^CD11c^+^CD138^+^ cells were significantly increased. And depletion, T-bet^+^ B cells reduced the percentage and absolute number of CD11c^+^ B cells and the levels of antichromatin IgG and IgG2a production. More recently, a report also showed that B cell-specific deletion of T-bet reduced the titers of autoantibodies and the appearance of CD11c^+^ B cells in SLE mice [49]. These data suggest that T-bet might regulate CD11c^+^ B-cell differentiation and activation in the development of lupus. However, the underlying mechanism of T-bet in the development and function of CD11c^+^ B cells is still unclear, and needs to be further explored.

IFNγ has been reported to play a pathogenic role in lupus nephritis. Depletion of IFNγ significantly improved renal disease induced by pristane, with reduction in the production of the anti-DNA/chromatin autoantibody [50–52]. Treatment of MRL-Lpr mice with IFNγR/Fc significantly reduced serum levels of IFNγ and autoantibody, consequently improving renal pathology. IFNγ is well known to be able to induce B cell IgG2a class-switching [53]. An in vitro study showed that IFNγ and anti-IgM synergistically induced T-bet expression in B cell in a STAT-1-dependent manner to promote IgG2a production [34]. However, it is still unclear how IFNγ triggers B cells to secrete autoantibodies in vivo. We noted that the expression of IFNγ was dramatically elevated both in the total spleen and in CD4^+^ T cells at day 14 post-cGVHD induction (Additional file 1: Figure S3A). IFNγ^+^CD4^+^ T cells were significantly increased in spleens from mice that received Bm12 splenocytes compared with the control group (Additional file 1: Figure S3B). Notably, the percentage and absolute number of T-bet^+^CD11c^+^CD19^+^ B cells was decreased in IFNGR1^–/–^ mice induced by cGVHD (Additional file 1: Figure S3C). Thus, we suggest that IFNγ might induce the expression of T-bet in B cells leading to their differentiation into CD11c^+^ B cells in lupus-like cGVHD mice. Further studies are needed to confirm this hypothesis.

A recent study showed that CD11c^+^ B cells are expanded only in a handful of SLE patients [21]. Others studies have shown that a population of B cells increased in the peripheral blood of rheumatoid arthritis (RA) patients and some autoimmune individuals with common variable immunodeficiency [54–56]. We found that the T-bet^+^CD11c^+^CD19^+^ B-cell population was significantly increased in the peripheral blood of SLE patients compared with healthy controls. Interestingly, the percentage of T-bet^+^CD11c^+^CD19^+^ B cells in PBMCs was associated with the sera level of antichromatin autoantibody, and elevated in SLE patients with lupus nephritis. Through further analysis, we found that the percentage of T-bet^+^CD11c^+^CD19^+^ B cells might be higher in patients with moderate or severe disease than in those with inactive disease (data not shown). These data suggest that T-bet^+^CD11c^+^CD19^+^ B cells might serve as a biomarker for clinical diagnosis and treatment for lupus patients. More clinical data are needed to verify this point.

## Conclusions

We have demonstrated that a newly discovered population of B cells, T-bet^+^CD11c^+^CD19^+^, is associated with the titer of antichromatin in lupus patients and is directly involved in secretion of autoantibody in cGVHD mice. Targeted depletion of CD11c or T-bet efficiently reduced the production of antichromatin autoantibodies. These findings highlight that T-bet^+^CD11c^+^CD19^+^ B cells might serve as a potential therapeutic target of lupus.

## Additional file

Additional file 1: T-bet^+^ CD11c^+^ B cells are critical for antichromatin immunoglobulin G production in the development of lupus. **Figure S1.** The fraction of T-bet and CD11c positive population was increased in CD138^+^ plasma cells. **Figure S2.** Correlation between the percentage of T-bet^+^ CD11c^+^ CD19^+^ B cells and the titers of anti-ANA antibodies (A), anti-dsDNA antibodies (B) from 22 SLE patients. **Figure S3.** IFNγ is required for T-bet^+^ CD11c^+^ B cell differentiation and activation. 5 × 10^7^ splenocytes from Bm12 mice or B6 mice cells were injected intraperitoneally into B6 mice (*n* = 5). (PDF 361 kb)

## Abbreviations

ACR: American College of Rheumatology
ANA: Antinuclear antibody
C1q: Complement 1q
CCHMC: Cincinnati Children’s Hospital Medical Center
cGVHD: Chronic graft-versus-host disease
Dnase I: Deoxyribonuclease I
dsDNA: Double-stranded DNA
ELISA: Enzyme-linked immunosorbent assay
HRP: Horseradish peroxidase
IC: Immunoglobulin complex
IFN: Interferon
Ig: Immunoglobulin
IHS: Institute of Health Science
LPS: Lipopolysaccharide
mAbs: Monoclonal antibodies
MHC: Major histocompatibility complex
PBMC: Peripheral blood mononuclear cell
PBS: Phosphate-buffered saline
qPCR: Quantitative polymerase chain reaction
RA: Rheumatoid arthritis
RNP: Ribonucleoprotein
RT: Room temperature
SAP: Serum amyloid protein
SD: Standard deviation
SLE: Systemic lupus erythematosus
SLEDAI: Systemic Lupus Erythematosus Disease Activity
TLR: Toll-like receptor
TMB: Tetramethylbenzidine

## References

[1] Mok CC, Lau CS (2003). Pathogenesis of systemic lupus erythematosus. J Clin Pathol..

[2] Rahman A, Isenberg DA (2008). Systemic lupus erythematosus. N Engl J Med..

[3] Sherer Y, Gorstein A, Fritzler MJ, Shoenfeld Y (2004). Autoantibody explosion in systemic lupus erythematosus: more than 100 different antibodies found in SLE patients. Semin Arthritis Rheum..

[4] Olsen NJ, Karp DR (2014). Autoantibodies and SLE: the threshold for disease. Nat Rev Rheumatol.

[5] Li J, Leng X, Li Z, Ye Z, Li C, Li X (2014). Chinese SLE treatment and research group registry: III. Association of autoantibodies with clinical manifestations in Chinese patients with systemic lupus erythematosus. J Immunol Res..

[6] Gharavi AE, Harris EN, Asherson RA, Hughes GR (1987). Anticardiolipin antibodies: isotype distribution and phospholipid specificity. Ann Rheum Dis.

[7] Van Venrooij WJ, Sillekens PT (1989). Small nuclear RNA associated proteins: autoantigens in connective tissue diseases. Clin Exp Rheumatol.

[8] Deshmukh US, Bagavant H, Fu SM (2006). Role of anti-DNA antibodies in the pathogenesis of lupus nephritis. Autoimmun Rev..

[9] Gomez-Puerta JA, Burlingame RW, Cervera R (2008). Anti-chromatin (anti-nucleosome) antibodies: diagnostic and clinical value. Autoimmun Rev..

[10] Cervera R, Viñas O, Ramos-Casals M, Font J, García-Carrasco M, Sisó A (2003). Anti-chromatin antibodies in systemic lupus erythematosus: a useful marker for lupus nephropathy. Ann Rheum Dis..

[11] Manson JJ, Ma A, Rogers P, Mason LJ, Berden JH, van der Vlag J (2009). Relationship between anti-dsDNA, anti-nucleosome and anti-alpha-actinin antibodies and markers of renal disease in patients with lupus nephritis: a prospective longitudinal study. Arthritis Res Ther..

[12] Hammady MR, Salam RF, Nabeh M (2011). Anti chromatin antibodies as a marker of lupus activity and lupus nephritis. Int J Acad Res..

[13] Morel L, Blenman KR, Croker BP, Wakeland EK (2001). The major murine systemic lupus erythematosus susceptibility locus, Sle1, is a cluster of functionally related genes. Proc Natl Acad Sci U S A..

[14] Li H, Zhang YY, Sun YN, Huang XY, Jia YF, Li D (2004). Induction of systemic lupus erythematosus syndrome in BALB/c mice by immunization with active chromatin. Acta Pharmacol Sin..

[15] Gomez-Puerta JA, Burlingame RW, Cervera R (2006). Anti-chromatin (anti-nucleosome) antibodies. Lupus..

[16] Dieker JW, van der Vlag J, Berden JH (2002). Triggers for anti-chromatin autoantibody production in SLE. Lupus..

[17] Eisenberg RA, Via CS (2012). T cells, murine chronic graft-versus-host disease and autoimmunity. J Autoimmun..

[18] Kootstra CJ, Van Der Giezen DM, Van Krieken JH, De Heer E, Bruijn JA (1997). Effective treatment of experimental lupus nephritis by combined administration of anti-CD11a and anti-CD54 antibodies. Clin Exp Immunol..

[19] Morris SC, Cheek RL, Cohen PL, Eisenberg RA (1990). Allotype-specific immunoregulation of autoantibody production by host B cells in chronic graft-versus host disease. J Immunol..

[20] Morris SC, Cohen PL, Eisenberg RA (1990). Experimental induction of systemic lupus erythematosus by recognition of foreign Ia. Clin Immunol Immunopathol..

[21] Rubtsov AV, Rubtsova K, Fischer A, Meehan RT, Gillis JZ, Kappler JW (2011). Toll-like receptor 7 (TLR7)-driven accumulation of a novel CD11c(+) B-cell population is important for the development of autoimmunity. Blood..

[22] Gilboe IM, Husby G (1999). Application of the 1982 revised criteria for the classification of systemic lupus erythematosus on a cohort of 346 Norwegian patients with connective tissue disease. Scand J Rheumatol..

[23] Dooley MA, Aranow C, Ginzler EM (2004). Review of ACR renal criteria in systemic lupus erythematosus. Lupus..

[24] Bombardier C, Gladman DD, Urowitz MB, Caron D, Chang CH (1992). Derivation of the SLEDAI. A disease activity index for lupus patients. The Committee on Prognosis Studies in SLE. Arthritis Rheum.

[25] McIntyre KR, Seidman JG (1984). Nucleotide sequence of mutant I-A beta bm12 gene is evidence for genetic exchange between mouse immune response genes. Nature..

[26] Seavey MM, Lu LD, Stump KL (2011). Animal models of systemic lupus erythematosus (SLE) and ex vivo assay design for drug discovery. Curr Protoc Pharmacol.

[27] Cohen PL, Maldonado MA (2003). Animal models for SLE. Curr protoc Immunol.

[28] Nimmerjahn F, Bruhns P, Horiuchi K, Ravetch JV (2005). FcgammaRIV: a novel FcR with distinct IgG subclass specificity. Immunity..

[29] Markine-Goriaynoff D, Coutelier JP (2002). Increased efficacy of the immunoglobulin G2a subclass in antibody-mediated protection against lactate dehydrogenase-elevating virus-induced polioencephalomyelitis revealed with switch mutants. J Virol..

[30] Boes M, Schmidt T, Linkemann K, Beaudette BC, Marshak-Rothstein A, Chen J (2000). Accelerated development of IgG autoantibodies and autoimmune disease in the absence of secreted IgM. Proc Natl Acad Sci U S A..

[31] Ehlers M, Fukuyama H, McGaha TL, Aderem A, Ravetch JV (2006). TLR9/MyD88 signaling is required for class switching to pathogenic IgG2a and 2b autoantibodies in SLE. J Exp Med..

[32] Gerth AJ, Lin L, Peng SL (2003). T-bet regulates T-independent IgG2a class switching. Int Immunol..

[33] Peng SL, Szabo SJ, Glimcher LH (2002). T-bet regulates IgG class switching and pathogenic autoantibody production. Proc Natl Acad Sci U S A..

[34] Xu W, Zhang JJ (2005). Stat1-dependent synergistic activation of T-bet for IgG2a production during early stage of B cell activation. J Immunol..

[35] Rubtsova K, Rubtsov AV, van Dyk LF, Kappler JW, Marrack P (2013). T-box transcription factor T-bet, a key player in a unique type of B-cell activation essential for effective viral clearance. Proc Natl Acad Sci U S A..

[36] Morris SC, Cheek RL, Cohen PL, Eisenberg RA (1990). Autoantibodies in chronic graft versus host result from cognate T-B interactions. J Exp Med..

[37] Shao WH, Gamero AM, Zhen Y, Lobue MJ, Priest SO, Albandar HJ (2015). Stat1 regulates lupus-like chronic graft-versus-host disease severity via interactions with Stat3. J Immunol..

[38] Eisenberg R (2003). The chronic graft-versus-host model of systemic autoimmunity. Curr Dir Autoimmun..

[39] Ceppellini R, Polli E, Celada F (1957). A DNA-reacting factor in serum of a patient with lupus erythematosus diffusus. Proc Soc Exp Biol Med..

[40] Cozzani E, Drosera M, Gasparini G (2014). Serology of lupus erythematosus: correlation between immunopathological features and clinical aspects. Autoimmune Dis..

[41] Yu C, Gershwin ME, Chang C (2014). Diagnostic criteria for systemic lupus erythematosus: a critical review. J Autoimmun..

[42] Chabre H, Amoura Z, Piette JC, Godeau P, Bach JF, Koutouzov S (1995). Presence of nucleosome-restricted antibodies in patients with systemic lupus erythematosus. Arthritis Rheum..

[43] Burlingame RW, Rubin RL, Balderas RS, Theofilopoulos AN (1993). Genesis and evolution of antichromatin autoantibodies in murine lupus implicates T-dependent immunization with self antigen. J Clin Invest..

[44] Xu Z, Vallurupalli A, Fuhrman C, Ostrov D, Morel L (2011). A New Zealand Black-derived locus suppresses chronic graft-versus-host disease and autoantibody production through nonlymphoid bone marrow-derived cells. J Immunol..

[45] Wang NS, McHeyzer-Williams LJ, Okitsu SL, Burris TP, Reiner SL, McHeyzer-Williams MG (2012). Divergent transcriptional programming of class-specific B cell memory by T-bet and RORalpha. Nat Immunol..

[46] Blanco F, Kalsi J, Ravirajan CT, Speight P, Bradwell AR, Isenberg DA (1992). IgG subclasses in systemic lupus erythematosus and other autoimmune rheumatic diseases. Lupus..

[47] Savitsky DA, Yanai H, Tamura T, Taniguchi T, Honda K (2010). Contribution of IRF5 in B cells to the development of murine SLE-like disease through its transcriptional control of the IgG2a locus. Proc Natl Acad Sci U S A..

[48] Slack JH, Hang L, Barkley J, Fulton RJ, D'Hoostelaere L, Robinson A (1984). Isotypes of spontaneous and mitogen-induced autoantibodies in SLE-prone mice. J Immnol..

[49] Rubtsova K, Rubtsov AV, Thurman JM, Mennona JM, Kappler JW, Marrack P (2017). B cells expressing the transcription factor T-bet drive lupus-like autoimmunity. J Clin Invest..

[50] Haas C, Ryffel B, Le Hir M (1998). IFN-gamma receptor deletion prevents autoantibody production and glomerulonephritis in lupus-prone (NZB × NZW) F1 mice. J Immnol..

[51] Schwarting A, Wada T, Kinoshita K, Tesch G, Kelley VR (1998). IFN-gamma receptor signaling is essential for the initiation, acceleration, and destruction of autoimmune kidney disease in MRL-Fas (lpr) mice. J Immnol..

[52] Haas C, Ryffel B, Le Hir M (1997). IFN-gamma is essential for the development of autoimmune glomerulonephritis in MRL/Ipr mice. J Immunol..

[53] Snapper CM, Paul WE (1987). Interferon-gamma and B cell stimulatory factor-1 reciprocally regulate Ig isotype production. Science..

[54] Warnatz K, Wehr C, Dräger R, Schmidt S, Eibel H, Schlesier M, Peter HH (2002). Expansion of CD19 (hi) CD21 (lo/neg) B cells in common variable immunodeficiency (CVID) patients with autoimmune cytopenia. Immunobiology..

[55] Isnardi I, Ng YS, Menard L, Meyers G, Saadoun D, Srdanovic I (2010). Complement receptor 2/CD21− human naive B cells contain mostly autoreactive unresponsive clones. Immunobiology..

[56] Rakhmanov M, Keller B, Gutenberger S, Foerster C, Hoenig M, Driessen G (2009). Circulating CD21low B cells in common variable immunodeficiency resemble tissue homing, innate-like B cells. Proc Natl Acad Sci U S A..

