# Supplementary material for: T-bet+CD11c+ B cells are critical for antichromatin immunoglobulin G production in the development of lupus
Source: Arthritis Res Ther. 2017 Oct 5;19:225. doi: 10.1186/s13075-017-1438-2 (PMC5629756; doi:10.1186/s13075-017-1438-2)
Supplement: Additional file 1: — T-bet+ CD11c+ B cells are critical for antichromatin immunoglobulin G production in the development of lupus. Figure S1. The fraction of T-bet and CD11c positive population was increased in CD138+ plasma cells. Figure S2. Correlation between the percentage of T-bet+ CD11c+ CD19+ B cells and the titers of anti-ANA antibodies (A), anti-dsDNA antibodies (B) from 22 SLE patients. Figure S3. IFNγ is required for T-bet+ CD11c+ B cell differentiation and activation. 5 × 10^7^ splenocytes from Bm12 mice or B6 mice cells were injected intraperitoneally into B6 mice (n = 5). (PDF 361 kb) [file 13075_2017_1438_MOESM1_ESM.pdf]

Figure S1

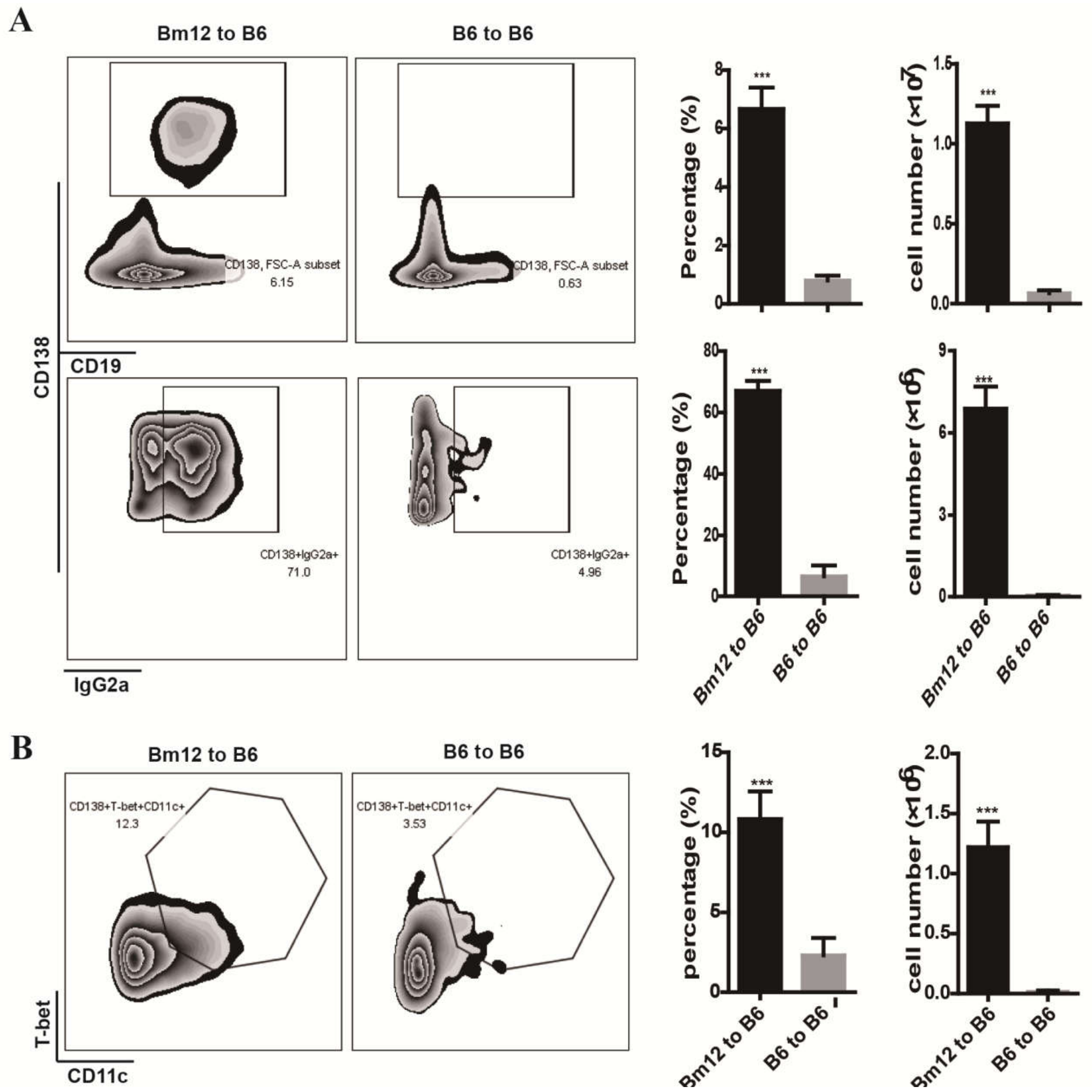

Figure S1. The fraction of T-bet and CD11c positive population was increased in CD138^{+} plasma cells. B6 mice (n=5) received an intraperitoneal injection of 5 × 10^7 splenocytes from Bm12 or B6. Spleens, collected at day 14, were analyzed for population of CD138^{+} cell and CD138^{+} IgG2a^{+} cell (A) and T-bet^{+} CD11c^{+} CD138^{+} cell (B). Bars represent the means ± SD for n = 5 mice per group. *** = P < 0.001.

Figure S2

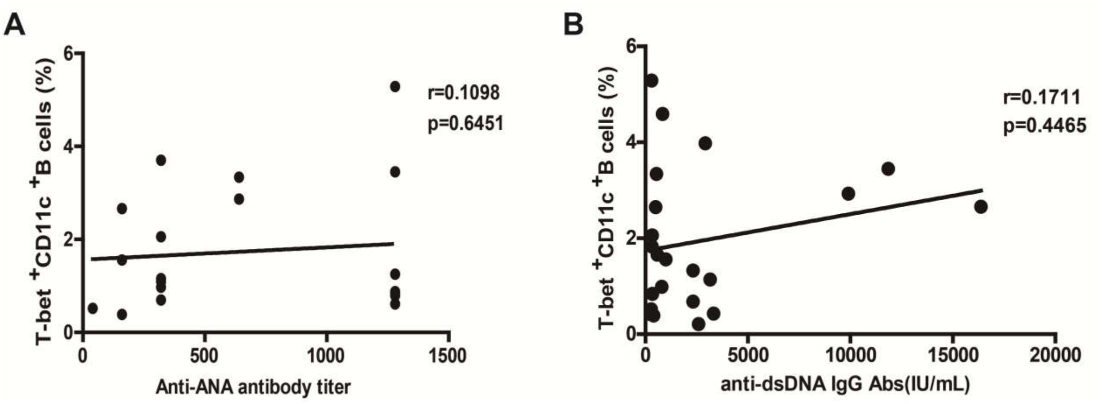

Figure S2. Correlation between the percentage of T-bet$^+$ CD11c$^+$ CD19$^+$ B cells and the titers of anti-ANA antibodies (A), anti-dsDNA antibodies (B) from 22 SLE patients. (Spearman's test was used for statistical analysis).

Figure S3

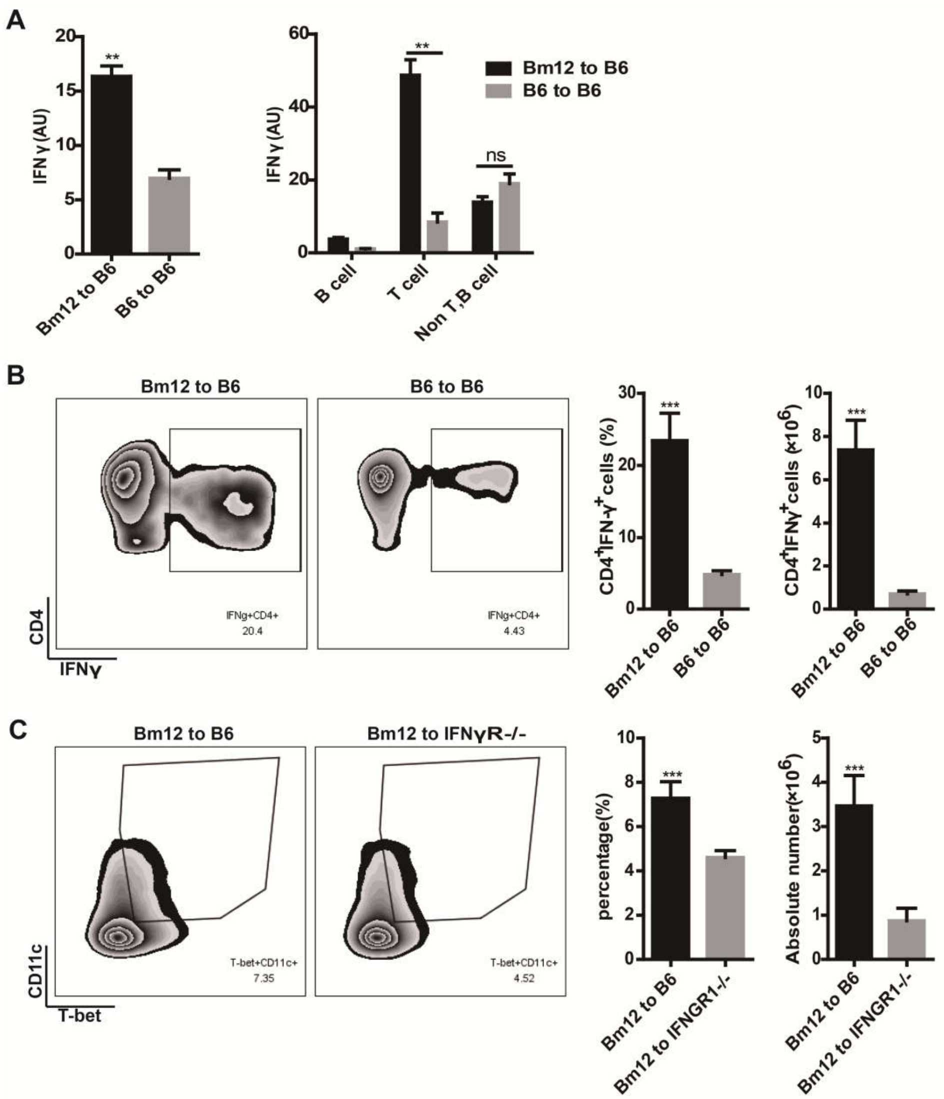

Figure S3. IFNγ is required for T-bet^{+} CD11c^{+} B cell differentiation and activation. 5×10^{7} Bm12 mice or B6 mice were injected intraperitoneally B6 mice (n = 5). After 14 days, spleen was collected for isolation of B cells, T cells and non-T and -B cells. IFNγ was detected by q-PCR. (A) IFNγ expression was tested in total splenocytes, B cells, T cells and non-T, B cells. (B) After 14 days, spleen T cells were isolated and cultured in vitro for 6 h in the presence of Golgistop and analyzed the expression of IFN-γ. (C) cGVHD model were induced in IFNGR1-/- mice. The figure shows that splenic T-bet^{+} CD11c^{+} CD19^{+} B cells are obviously reduced in the group from Bm12 to IFNGR-/- mice. Bars represent the means ± SD for n = 5 mice per group. ** = P < 0.01, *** = P < 0.001, ns = not significant.
